# Variant‐to‐function mapping of late‐onset Alzheimer’s disease GWAS loci in human microglial models implicates *RTFDC1* as an effector gene at the *CASS4* locus

**DOI:** 10.1002/alz.089683

**Published:** 2025-01-03

**Authors:** Elizabeth A Burton, Mariana Argenziano, Kieona Cook, Molly Ridler, Chun Su, Elisabetta Manduchi, Kenyaita M Hodge, Li‐San Wang, Gerard D. Schellenberg, James A. Pippin, Andrew D. Wells, Stewart A. Anderson, Christopher D. Brown, Struan F.A. Grant, Alessandra Chesi

**Affiliations:** ^1^ University of Pennsylvania, Philadelphia, PA USA; ^2^ The Children’s Hospital of Philadelphia, Philadelphia, PA USA; ^3^ The Children's Hospital of Philadelphia, Philadelphia, PA USA; ^4^ The University of Pennsylvania, Philadelphia, PA USA; ^5^ Emory University, Atlanta, GA USA

## Abstract

**Background:**

To date, Alzheimer’s disease (AD) research has principally focused on neurons. In contrast, recent studies suggest that genetic mechanisms drive microglia towards prolonged inflammation in AD brains, exacerbating neurodegeneration. Indeed, many of the 70 disease‐associated loci uncovered with genome‐wide association studies (GWAS) reside near genes related to microglial function, such as *TREM2*. However, GWAS cannot directly identify causal variants or effector genes, as GWAS only reports the sentinel variant at a given locus, which tags all variants in high linkage disequilibrium. We used a combination of ATAC‐seq and high‐resolution promoter‐focused Capture‐C in two human microglial cell models to map interactions between GWAS‐implicated variants and their putative effector genes. We then validated an observed interaction between SNP rs6024870 at the ‘*CASS4*’ AD locus and the promoter of *RTFDC1*, a DNA damage response gene, using CRISPR‐Cas9.

**Method:**

We employed Capture‐C to characterize the genome‐wide interactions of all promoters in the HMC3 microglial cell line and iPSC‐derived microglia. We confirmed enhancer activity of the region harboring rs6024870 using dual‐luciferase assays. To validate the regulatory interaction with the Capture‐C implicated effector gene *RTFDC1*, we used lentiviral CRISPR‐Cas9 to delete an ∼400bp region containing the SNP in HMC3 cells. We confirmed changes in RNA and protein expression using RNA‐seq and immunoblotting. Furthermore, we assessed DNA damage repair deficiencies in enhancer knockout cells using an immunofluorescent assay for the histone mark GH2AX, and confirmed the role of RTFDC1 in the DNA damage phenotype using siRNA knockdown and lentiviral overexpression of *RTFDC1*, respectively.

**Result:**

Proxy SNP, rs6024870 (r^2^ = 0.93 to sentinel rs6014724), at the ‘*CASS4*’ locus, coincided with open chromatin and contacted the promoter of *RTFDC1*. Deletion of the putative enhancer region harboring rs6024870 by CRISPR‐Cas9 in HMC3 reduced the expression of both *RTFDC1* mRNA and protein. Knockout cells had reduced ability to clear DNA double‐strand breaks (DSBs), and siRNA knockdown and lentiviral overexpression of *RTFDC1* confirmed this phenotype was due to reduction of *RTFDC1*.

**Conclusion:**

We implicate rs6024870 and *RTFDC1* as a SNP‐effector gene pair at the AD ‘*CASS4’* GWAS locus, and the gene product is directly involved in driving a reduction in DNA DSB clearance.

